# Factors influencing basic vaccination coverage in Myanmar: secondary analysis of 2015 Myanmar demographic and health survey data

**DOI:** 10.1186/s12889-019-6548-0

**Published:** 2019-02-28

**Authors:** Ikuma Nozaki, Masahiko Hachiya, Tomomi Kitamura

**Affiliations:** 1 Bureau of International Health Cooperation, National Center for Global Health and Medicine, 1-21-1, Toyama, Shinjuku, Tokyo, 162-8655 Japan; 2 Ministry of Health, Naypyidaw, Myanmar

**Keywords:** Childhood, Immunization, Coverage, Vaccines, Demographic health survey, Myanmar

## Abstract

**Background:**

Immunization is one of the most effective measures for preventing disease when vaccination coverage is sufficient. Although vaccination coverage is known to be influenced by social and cultural barriers, the determinants of childhood immunization in Myanmar remain poorly understood. This study analyzed factors that influenced complete vaccination status (one dose each for Bacillus Calmette-Guérin and measles and three doses each for diphtheria-pertussis and polio) using 2015 data from the Myanmar Demographic Health and Survey.

**Methods:**

Data from 12 to 23-month-old children and their mothers were extracted from the nationally representative survey results. Bivariate and multivariate analyses with survey-weighted logistic regression were performed to examine the relationships between vaccination status and various sociodemographic and medical factors. The independent variables for the analyses included area of residence, economic status, maternal age, marital status, education, literacy, employment status, antenatal care attendance, tetanus vaccination, place of delivery, postnatal evaluations, child’s sex, number of children, previous child death, decision maker(s) regarding child’s health, frequency of healthcare visits, paternal education, and paternal occupation.

**Results:**

A representative sample of 904 cases were extracted for the analysis. The overall complete vaccination rate was 55.4%. In the multivariate analysis with backward step-wise selection, complete vaccination was independently associated with middle or high economic status (adjusted odds ratio [AOR]: 2.64, 95% confidence interval [CI]: 1.85–3.78), older maternal age (AOR: 2.87, 95% CI: 1.62–5.10), ≥4 antenatal care visits (AOR: 1.87, 95% CI: 1.28–2.73), and maternal tetanus vaccination before delivery (AOR: 3.26, 95% CI: 1.82–5.85).

**Conclusion:**

The first Demographic and Health Survey in Myanmar revealed that only approximately one-half of 12–23-year-old children had received complete vaccination, which was lower than the estimated rate from routine administrative coverage. Our results indicate that incomplete immunization status was associated with low economic status, younger maternal age, fewer antenatal care visits, and no maternal tetanus vaccination. These findings may help improve the targeting and strategic implementation of the Expanded Programme on Immunization.

## Background

Immunization is one of the most successful and cost-effective tools for improving children’s health [1]. Furthermore, the public health community agrees that completing a full course of vaccinations against communicable diseases (e.g., measles, polio, and others) helps reduce morbidity and mortality among children. However, childhood vaccination rates vary between and within countries, with low- and middle-income countries remaining vulnerable to vaccine-preventable diseases [2]. In 1974, the World Health Organization launched the Expanded Programme on Immunization (EPI) to help globally control vaccine-preventable diseases [3]. In Myanmar, the EPI was launched in 1978 by introduction of Bacillus Calmette-Guérin (BCG), diphtheria-pertussis-tetanus (DPT), and tetanus toxoid (TT) vaccines [4]. Vaccines to prevent measles and polio were subsequently added onto the routine vaccination schedule in 1987. The coverage of EPI in Myanmar has gradually expanded from originally 104 of 330 townships at the beginning to 305 townships in 1995, with almost all areas of all townships thought to be covered [4]. As a result, the national immunization coverage rates in 2015 were 86% for BCG, 75% for DPT3, 76% for oral polio vaccine 3 (OPV3), and 86% for measles-containing-vaccine first-dose. Furthermore, 85% of the townships have achieved 80% coverage using pentavalent vaccines and 45% of the townships have achieved more than 90% coverage using measles-containing-vaccine first-dose [5].

The first Myanmar Demographic and Health Survey (MDHS) was conducted during 2015–2016, as part of the global Demographic and Health Survey (DHS) Program supported by USAID. It was a nationally representative household survey that used a standardized questionnaire with questions regarding vaccination coverage. The study sample was selected using a stratified two-stage cluster sampling design [6]. The MDHS 2015–16 reported that only 55% of 12–23-month-old children had received all basic vaccinations (one dose each for BCG and measles and three doses each for DP and polio), with a noticeable discrepancy between the comparatively high administrative coverage and the comparatively low MDHS-reported coverage. Furthermore, Myanmar has experienced outbreaks of vaccine-preventable diseases in 2015, 2016, and 2017 (e.g., measles, diphtheria, and Japanese encephalitis), even in areas with reportedly high administrative coverage. Moreover, two cases involving a circulating vaccine-derived polio virus were detected in 2015. Thus, there is a need to better understand the factors that can influence complete vaccination coverage to improve the situation in Myanmar.

Various factors can influence complete vaccination coverage, such as access to the vaccination site [7, 8], parental age and education [7–9], household income and wealth [8, 9], the child being a girl [10, 11], and place of delivery [9, 12]. In addition, Rainey et al. performed a systematic review of non-vaccination and under-vaccination among children in low- and middle-income countries, and proposed that these outcomes were related to the immunization system, family characteristics, parental attitudes and knowledge, and limitations regarding immunization-related communication and information [13]. Therefore, the present study aimed to identify factors that were associated with completion of all basic vaccinations in Myanmar, based on data from the 2015–2016 MDHS.

## Methods

### Study design and data source

The present study involved a secondary analysis of MDHS data (collected between December 2015 and July 2016). This was the first Myanmar survey as part of the worldwide DHS [6, 14], which aimed to provide policy makers and program managers with up-to-date estimates of basic demographic and health indicators in order to evaluate and develop programs and strategies [6]. A stratified two-stage clustered sampling design was used, with the first stage involving 442 clusters (123 urban and 319 rural clusters) from 30 sampling strata and the second stage involving 30 households from each cluster (i.e., 13,260 households) [6]. All women who were 15–49-years-old, and members of the selected household or visitors who stayed with the selected household the night before the survey, were considered eligible [14]. The selected and eligible women provided maternal and child-related data using the Women’s Questionnaire, which was developed for the worldwide DHS program and modified for the Myanmar survey based on its culture and specific health challenges [6]. Vaccination coverage was assessed using both the child’s vaccination card and the mother’s recollection (as only 45% of the target children had available vaccination cards).

The DHS Program produces various datasets, including household data, household economic status, women’s data, men’s data, couples’ data, child-related data, and birth-related data. The present study evaluated maternal and children’s data, which included records for each child who had been born to the interviewed woman in the 5 years before the survey (i.e., all children were 0–59 months old). These records contained information related to the mother, pregnancy, postnatal care, immunization, and health.

### Statistical analysis

Data regarding 12–23-month-old children were extracted from the dataset, and all analyses were performed using the survey feature of Stata software (version 14.0; StataCorp, College Station, TX). The study outcome was defined as complete vaccination status, which involved receiving one dose each for BCG and measles and three doses each for DPT and polio. We selected 18 variables from the DHS questionnaire that might potentially be associated with immunization status: area of residence, economic status, maternal age, marital status, maternal education, maternal literacy, maternal occupation, antenatal care (ANC) attendance, maternal tetanus vaccination, place of delivery, postnatal evaluation, child’s sex, number of children, previous child death, decision maker(s) regarding the child’s health, frequency of healthcare visits, paternal education, and paternal occupation. Bivariate analyses were performed to evaluate the relationships between vaccination status and the various factors using survey-weighted logistic regression. Multivariate analyses were subsequently performed using a backward step-wise logistic regression model. Multi-collinearity was tested and an acceptable result was defined as a variance inflation factor of < 10.

## Results

Children’s recode file provided by DHS program contained 4815 data of children under 5-year-old. The present study extracted and evaluated data from 904 mothers and their children who were 12–23 months old. Among these children, only 55.4% had complete vaccination coverage (one dose each for BCG and measles and three doses each for DP and polio). Table 1 shows that the rates of complete immunization coverage were 67.8% in urban settings and 50.9% in rural settings. According to maternal education, the complete coverage rates were 51.2% for no or primary education and 61.4% for secondary or higher education. According to economic status, the complete coverage rates were 41.4% for low economic status and 69.2% for middle or high economic status. According to maternal age, the complete coverage rates were 42.1% for 15–24 years old, 57.2% for 25–34 years old, and 63.9% for ≥35 years old. The complete coverage rates were 62.0% for home delivery and 49.4% for delivery in a healthcare setting. The complete coverage rates were 58.2% for male children and 51.7% for female children.

**Table 1:** Full vaccination coverage and related factors among 12–23-month-old children in Myanmar

	Weighted number of respondents	Full vaccination % (95% CI)	Crude OR (95% CI)	*p*-value
Area of residence				
Urban	207	67.8 (60.4–75.2)	REF	
Rural	697	50.9 (45.2–56.6)	0.493 (0.329–0.740)	0.001
Economic status				
Low	459	41.4 (34.9–47.8)	REF	
Middle or high	440	69.2 (63.9–74.5)	3.178 (2.259–4.470)	< 0.001
Maternal age				
15–24 years	207	42.1 (34.0–50.2)	REF	
25–34 years	474	57.2 (51.4–63.0)	1.828 (1.233–2.711)	0.003
≥ 35 years	214	63.9 (55.3–72.6)	2.503 (1.524–4.111)	< 0.001
Marital status				
Separated (any reason)	29	41.2 (15.4–66.9)	REF	
Married	868	55.8 (51.0–60.7)	1.608 (0.732–3.535)	0.236
Maternal education				
None/primary school	528	51.2 (45.0–57.5)	REF	
Secondary or higher	374	61.4 (56.0–66.9)	1.515 (1.097–2.091)	0.012
Maternal literacy				
Cannot read sentences	245	45.1 (35.8–54.4)	REF	
Can read sentences	654	58.9 (54.4–63.3)	1.681 (1.137–2.485)	0.009
Maternal employment				
Unemployed	361	55.6 (48.2–62.9)	REF	
Unskilled employee	328	49.9 (42.9–57.0)	0.807 (0.543–1.198)	0.286
Skilled employee	207	62.2 (53.5–70.9)	1.316 (0.841–2.058)	0.228
ANC attendance				
0–3 times	341	37.7 (30.2–45.1)	REF	
≥ 4 times	554	65.4 (60.6–70.3)	3.012 (2.093–4.335)	< 0.001
Tetanus vaccination				
No	89	25.9 (14.8–37.1)	REF	
Once or more	803	58.7 (54.3–63.1)	3.677 (1.985–6.809)	< 0.001
Place of birthing				
Healthcare setting	523	49.4 (42.9–55.9)	REF	
Home	377	62.0 (56.3–67.6)	1.667 (1.185–2.326)	0.003
Postnatal evaluation				
Not within 2 months	453	53.0 (47.3–58.7)	REF	
Yes	412	57.8 (51.6–63.9)	1.202 (0.877–1.649)	0.252
Child’s sex				
Male	499	58.2 (52.3–64.2)	REF	
Female	404	51.7 (45.6–57.8)	0.764 (0.550–1.061)	0.108
Number of children				
1	501	57.5 (51.5–63.6)	REF	
≥ 2	401	52.0 (46.0–58.0)	0.800 (0.576–1.111)	0.182
Prior child death				
Never	775	57.0 (52.2–61.8)	REF	
Ever	123	43.4 (29.6–57.2)	0.566 (0.342–0.935	0.026
Child health decisions				
Mother alone	502	57.9 (52.2–63.5)	REF	
Mother + other(s)	400	51.9 (45.8–58.1)	0.789 (0.580–1.074)	0.131
Healthcare visits				
Not in last 12 months	421	49.0 (42.4–55.7)	REF	
Visit in last 12 months	482	60.5 (54.8–66.2)	1.589 (1.127–2.241	0.008
Paternal education				
None/primary school	493	47.2 (41.0–53.5)	REF	
Secondary or higher	407	65.3 (59.5–71.2)	2.101 (1.489–2.965)	< 0.001
Paternal occupation				
Unskilled employee	586	50.8 (45.2–56.5)	REF	
Skilled employee	312	62.6 (55.2–70.0)	1.597 (1.098–2.322)	0.014

*CI*: confidence interval, *OR*: odds ratio, *REF*: reference category, *ANC* antenatal care

In the bivariate analysis, complete vaccination coverage was associated with urban residence (odds ratio [OR]: 2.03, 95% confidence interval [CI]: 1.35–3.04), middle or high economic status (OR: 3.18, 95% CI: 2.26–4.47), older maternal age (OR: 2.50, 95% CI: 1.52–4.11), greater maternal education (OR: 1.52, 95% CI: 1.10–2.09), maternal literacy (OR: 1.68, 95% CI: 1.14–2.49), ≥4 ANC visits (OR: 3.01, 95% CI: 2.09–4.34), maternal tetanus vaccination before delivery (OR: 3.68, 95% CI: 1.99–6.81), home birth (OR: 1.67, 95% CI: 1.19–2.33), no previous child death in the household (OR: 1.77, 95% CI: 1.07–2.92), visiting a healthcare facility during the last 12 months (OR: 1.59, 95% CI: 1.13–2.24), greater paternal education (OR: 2.10, 95% CI: 1.49–2.97), and skilled paternal occupation (OR: 1.60, 95% CI: 1.10–2.32) (Table 1).

The multivariate analysis with backward step-wise selection revealed that complete vaccination coverage remained independently associated with middle or high economic status (adjusted OR [AOR]: 2.64, 95% CI: 1.85–3.78), older maternal age (AOR: 2.87, 95% CI: 1.62–5.10), ≥4 ANC visits (AOR: 1.87, 95% CI: 1.28–2.73), and maternal tetanus vaccination before delivery (AOR: 3.26, 95% CI: 1.82–5.85). The final model also included no previous child death in the household, although the results were not significant (AOR: 1.60, 95% CI: 0.92–2.78) (Table 2).

**Table 2:** Multivariable logistic regression analysis of full immunization coverage among 12–23-month-old children

	Adjusted OR (95% CI)	*p*-value
Economic status		
Low	REF	
Middle or high	2.642 (1.848–3.778)	< 0.001
Maternal age		
15–24 years	REF	
25–34 years	1.895 (1.227–2.926)	0.004
≥ 35 years	2.870 (1.620–5.102)	< 0.001
ANC attendance		
0–3 times	REF	
≥ 4 times	1.871 (1.283–2.727)	0.001
Tetanus vaccination		
No	REF	
Once or more	3.260 (1.816–5.851)	< 0.001
Prior child death		
Ever	REF	
Never	1.600 (0.921–2.778)	0.095

*CI* confidence interval, *OR* odds ratio, *REF*: reference category, *ANC* antenatal care

## Discussion

Although the benefits of vaccination are widely recognized, a global estimate indicated that 19,500,000 infants in 2016 were not covered by routine immunization services, such as the DTP3 vaccine [15]. Moreover, despite Myanmar’s remarkable progress in early vaccination efforts, the rate of complete coverage has not been improving during recent years [4]. For example, the MDHS data revealed that only 55% of 12–23-month-old children had complete vaccination coverage, which is lower than the WHO-UNICEF estimates of routine administrative coverage from 2014, such as 86% coverage for BCG, 75% coverage for DTP3, 76% coverage for OPV3, and 86% coverage for measles-containing-vaccine first-dose [16]. Although the population coverage required to achieve herd immunity varies according to disease, the ideal target is 100% coverage because vaccination coverage is not a perfect measure of population-level immunity. One author has described this concept as “everyone who can get vaccinated, should get vaccinated – not only to protect themselves, but to protect those who can’t, through herd immunity” [17]. Therefore, we evaluated factors that were related to complete vaccination coverage in Myanmar, and found that complete coverage was independently associated with higher economic status, older maternal age, ≥4 ANC visits, maternal tetanus vaccination before delivery, and no previous child death in the household.

Because socioeconomic status is known to be associated with vaccination coverage [18–23], we used the DHS wealth index to assess household socioeconomic status. This index provides a composite measure of a household’s cumulative living standard, which is categorized into 5 wealth quintiles and is used to quantify whether household economic status affects health outcomes. The wealth index is calculated based on easily collected data regarding select assets, such as televisions, bicycles, and materials used for housing construction, and types of water access and sanitation facilities, because reliable data regarding income and expenditures (i.e., the traditional indicators of household economic status) are not available in many countries [24]. The present study revealed a strong association between higher economic status and full vaccination coverage. However, this relationship may also be confounded by other well-known predictors of complete vaccination coverage, such as maternal education and literacy or the area of residence (urban or rural) [25, 26]. The present study revealed strong associations between the wealth index and maternal education (*p* < 0.001), maternal literacy (*p* < 0.001), and area of residence (*p* < 0.001). Although these factors were significant in the bivariate analyses, they were no significant in the multivariate analyses. In contrast, older maternal age remained an independent predictor in the multivariate analysis. Interestingly, previous studies have indicated that maternal age was both negatively and positively correlated with complete vaccination coverage [22, 27, 28].

Child sex and the number of children in the household are also well-known predictors of vaccination coverage in some social contexts [10, 11, 29]. However, these factors were not significant in Myanmar, although we did observe slightly higher complete vaccination coverage rates among male children (58% vs. 52%) and single children versus households with ≥2 children (58% vs. 52%). Maternal empowerment may also influence vaccination coverage in some countries [30–32]. Thus, we evaluated the complete vaccination coverage rates for households where the mother made all health-related decisions and households where the mother had to consult other people, although we failed to detect a significant difference between these two groups.

Several studies have indicated that complete immunization coverage is associated with various maternal healthcare utilization indices, such as ANC attendance, pre-birth TT immunization, skilled birth attendance, and delivery in a healthcare facility [33–36]. Danchin et al. have reported that vaccination decision making begins during pregnancy [37], and we also found that complete vaccination coverage was associated with maternal ANC attendance and pre-birth TT immunization. However, we found that complete vaccination coverage was less common for births at a healthcare facility, relative to home births, although this difference was not significant in the multivariate analysis. This is likely related to the relationship between ANC attendance and home birthing (*p* < 0.001), although the cause of this relationship remains unclear and further research is needed to better understand why Myanmar women would select a home birth if they frequently attended ANC visits.

During the planning phase of this study, we assumed that households who had previously experienced a child’s death might be more likely to vaccinate subsequent children, relative to households who had not experienced a child’s death. However, we were surprised to find that households with a previous child’s death had a lower complete vaccination rate. Nevertheless, households with a previous child’s death had a relatively low economic status (*p* < 0.001), which also strongly predicts incomplete immunization coverage. Therefore, a previous child’s death may be related to the household’s low economic status, less attention paid to the child’s health, and incomplete vaccination. Vaccination to child’s death, vaccination avoidance due to misunderstandings relating vaccination to previous child’s death could also be possible explanation for this. Further studies are needed to understand the healthcare perspectives of caretakers who have lost a child, as healthcare workers and health officials may need to use a different approach for these individuals. As an alternate indirect index for healthcare access, we evaluated whether the mother had visited a healthcare facility during the last 12 months. Similar to a previous report [38], we found a significant association between a recent healthcare visit and complete vaccination, although this relationship was not significant in the multivariate analysis. Therefore, further studies are needed to better understand the barriers to healthcare access in Myanmar.

The present study has several limitations. For example, only extracting data for 12–23-month-old children may have introduced bias (the sample unit for the 2015–2016 MDHS was households). Since vaccination coverage was assessed using both the child’s vaccination card and the mother’s recollection according to WHO recommendations, there were potential risk of recall bias and subject bias that mothers of children who don’t have the vaccination card tend to report a vaccination for their children due to shame and stigma [38]. Furthermore, the DHS is not designed to assess the EPI program status, as it does not consider factors that can influence immunization systems, such as access and distance from the vaccination services, missed vaccination opportunities, and limited availability and knowledge of health workers [13]. Although these factors are known to affect vaccine coverage, the present study was only able to evaluate them using indirect measures (e.g., healthcare facility use within the past year and area of residence). The present study also failed to evaluate regional variations in complete vaccination coverage, as the small number of children in each state/region would have produced a low statistical power. Moreover, other countries have had discrepancies between the vaccination coverage rate and population-level immunity for vaccine-preventable diseases, which may be related to insufficient cold chain control and the vaccine’s heat sensitivity [39]. Thus, to evaluate the risks of related outbreaks, direct immunity assessment is needed (e.g., seroepidemiological analysis). Therefore, combining the DHS with serological assessments would greatly improve our ability to understand regional differences and discrepancies between the vaccination coverage rate and population-level immunity, as the DHS data are representative of the region’s general population. Despite these limitations, secondary analysis of DHS data is thought to substantially contribute to public health knowledge in developing countries [40, 41], and we believe that our findings can help inform decisions regarding related policies and programs.

## Conclusion

The first MDHS revealed a complete vaccination coverage rate of only 55% among 12–23-month-old children, which is lower than the estimated rate from routine administrative coverage. The present study also revealed that incomplete vaccination coverage was independently associated with low economic status, younger maternal age, fewer ANC visits, and no pre-birth maternal tetanus vaccination. These findings may help the EPI program more specifically target and better address these demographic groups. However, further studies are needed to understand the relationships between maternal healthcare utilization (e.g., birthing location), immunization system-related factors (e.g., access and distance from the vaccination services), and population-level immunity.

## Abbreviations

ANC: antenatal care
BCG: Bacillus Calmette-Guérin
CI: confidence interval
DHS: Demographic and Health Survey
DPT: diphtheria-pertussis-tetanus
EPI: The Expanded Program on Immunization
OPV: oral polio vaccine
OR: odds ratio
TT: tetanus toxoid
